# Real‐Time Invasive Hemodynamic Changes During Endobronchial Valve Therapy in Advanced Emphysema: A Prospective Case Series

**DOI:** 10.1002/pul2.70451

**Published:** 2026-11-05

**Authors:** Ciaran Grafton‐Clarke, James Harrison, Della Mantle, Gareth Matthews, Pankaj Garg, Vasileios Kouritas, Henry O'Connor

**Affiliations:** ^1^ School of Medicine University of East Anglia Norwich UK; ^2^ Department of Cardiology Norfolk and Norwich University Hospitals NHS Foundation Trust Norwich UK; ^3^ Department of Anaesthetics and Critical Care Norfolk and Norwich University Hospitals NHS Foundation Trust Norwich UK; ^4^ Department of Thoracic Surgery Norfolk and Norwich University Hospitals NHS Foundation Trust Norwich UK

**Keywords:** emphysema, endobronchial valve, invasive hemodynamic assessment, pulmonary hypertension

## Abstract

Pulmonary hypertension (PH) in advanced emphysema is associated with worse outcomes and often prompts caution or exclusion from bronchoscopic lung volume reduction. The immediate pulmonary hemodynamic response to endobronchial valve (EBV) deployment has not been characterized using invasive measurements obtained during implantation. We performed a prospective single‐center physiological case series of four consecutive patients undergoing clinically indicated EBV therapy with concurrent right heart catheterization and continuous systemic arterial pressure monitoring. Hemodynamics were sampled at end‐expiration at four predefined timepoints: pre‐general anesthesia (pre‐GA), GA pre‐valve, GA post‐valve, and post‐extubation once awake and hemodynamically stable. Cardiac output was estimated by the indirect Fick method using an assumed oxygen consumption. All patients met hemodynamic criteria for PH with a pre‐capillary profile at baseline. In the prespecified GA comparison (pre‐valve to post‐valve), mean pulmonary artery pressure (mPAP) decreased in all four patients (−10% to −61%). In the prespecified awake comparison (pre‐GA to post‐extubation), mPAP decreased in all four (−33% to −58%), with post‐extubation mPAP < 20 mmHg in three patients. Estimated pulmonary vascular resistance decreased in three under GA (−42% to −90%) and in all four in the awake comparison (−47% to −96%); pulmonary arterial compliance increased in all four. Real‐time invasive hemodynamic assessment during EBV implantation was feasible in this four‐patient case series and showed descriptive, hypothesis‐generating reductions in pulmonary vascular load in selected patients with advanced emphysema and baseline pre‐capillary PH.

## 1 Introduction

Pulmonary hypertension (PH) is a common and prognostically adverse complication of advanced emphysema [1]. In this context, elevated pulmonary artery (PA) pressures are frequently taken to reflect advanced pulmonary vascular disease and adverse cardiopulmonary interaction, and are associated with reduced exercise capacity, right ventricular (RV) dysfunction, and increased peri‐procedural risk [2, 3]. As a result, suspected or established PH often shifts decision‐making away from lung‐volume‐reduction interventions toward conservative management [4].

Endobronchial one‐way valves (EBVs) are an established bronchoscopic lung volume reduction therapy for carefully selected patients with severe emphysema and marked hyperinflation, with randomized trials demonstrating improvements in lung mechanics, exercise capacity, and health‐related quality of life [5, 6, 7, 8]. However, patients with PH have been under‐represented in the pivotal evidence base. Major EBV trials generally excluded significant PH [9, 10], and contemporary expert guidance recommends caution when PH is present and avoidance when it is severe [11, 12]. Accordingly, the pulmonary hemodynamic effects of EBV therapy in advanced emphysema with co‐existing PH remain poorly characterized.

Pulmonary vascular load in advanced emphysema reflects not only fixed structural pulmonary vascular disease but may also include mechanically and hypoxically mediated components related to hyperinflation and ventilation–perfusion mismatch [13]. By initiating regional deflation of a targeted hyperinflated lobe, EBV therapy could plausibly reduce these contributors and produce hemodynamic unloading within the procedural window [14]. Despite this physiological rationale, direct invasive evidence capturing the immediate hemodynamic response at the time of valve deployment remains limited.

In this prospective physiological case series, we performed real‐time right heart catheterization (RHC) in high‐risk patients with advanced emphysema undergoing clinically indicated EBV therapy, acquiring paired invasive hemodynamic measurements immediately before and after deployment. Predefined acquisitions before induction and under stable general anesthesia (GA) were used to distinguish state‐related changes from valve‐associated changes as far as possible in this design, describe the immediate pulmonary vascular response, and generate hypotheses regarding hemodynamic lability in a subgroup often considered high risk for intervention.

## 2 Methods

### 2.1 Study Design and Setting

This was a prospective single‐center physiological case series in patients with advanced emphysema undergoing endobronchial valve therapy.

### 2.2 Participants

Consecutive patients with advanced emphysema undergoing clinically indicated endobronchial valve therapy were included. No additional inclusion or exclusion criteria were applied beyond routine clinical eligibility for valve treatment within the institutional emphysema service. Patients underwent standard multidisciplinary assessment, including physiological testing and imaging, comprising thin‐slice (1 mm) computerized tomography (CT) with quantitative analysis (StratX), perfusion imaging (SPECT), and transthoracic echocardiography (TTE) as part of routine pre‐procedural assessment. All patients provided written informed consent.

### 2.3 Endobronchial Valve Procedure

Valve implantation was performed in the operating theater using flexible bronchoscopy under GA with endotracheal intubation. The Zephr one‐way valve system (Pulmonx, Redwood City, CA, USA) was used. Target lobe selection was determined through multidisciplinary review incorporating pre‐procedural imaging and physiological assessment. Collateral ventilation (CV) status was confirmed using the Chartis Pulmonary Assessment System (Pulmonx, Redwood City, CA, USA). Valves were deployed into the segmental and subsegmental bronchi of the target lobe with the aim of achieving complete lobar occlusion. Valves were repositioned or exchanged where necessary to achieve an adequate seal.

### 2.4 Anesthesia and Ventilatory Management

Procedures were performed under GA with controlled mechanical ventilation. Anesthesia followed a consistent total intravenous technique (propofol and alfentanil) with neuromuscular blockade (atracurium). FiO_2_ (0.5–0.7) and ventilator settings were kept constant within each patient between the paired intra‐anesthetic pre‐ and post‐valve hemodynamic acquisitions to minimize ventilatory confounding of pulmonary vascular tone, although this could not eliminate the physiological effects of GA and controlled ventilation (Supporting Information S1: Tables 1 and 2).

### 2.5 Right‐Heart Catheterization Protocol

RHC was performed via ultrasound‐guided left basilic venous access using a 6 Fr introducer. A balloon‐tipped, fluid‐filled PA catheter (Swan‐Ganz True Size Monitoring Catheter, Double Lumen; Edwards Lifesciences, Irvine, CA, USA) was advanced under pressure waveform guidance to obtain sequential recordings in the right atrium (RA), RV, PA, and pulmonary capillary wedge position (PCWP). Transducers were flushed, zeroed to atmospheric pressure, and levelled at the mid‐axillary line prior to each acquisition. Continuous systemic arterial pressure was recorded concurrently via a right radial arterial line.

Pressures were sampled at end‐expiration. Awake measurements were obtained during a brief end‐expiratory breath‐hold. Measurements under GA were obtained at end‐expiration during controlled ventilation. Values were derived from at least three consecutive beats with stable waveform morphology. Wedge position was accepted when a typical wedge waveform was obtained. FiO_2_, ventilator settings, vasoactive support, and other procedural variables were kept as constant as clinically feasible between paired acquisitions, and measurements were recorded only once hemodynamics were stable.

### 2.6 Timing of Hemodynamic Measurements

Hemodynamic measurements were obtained at four predefined peri‐procedural timepoints. The first was before induction during spontaneous breathing. The second was after induction and endotracheal intubation, once controlled ventilation was established, and before any bronchoscopic assessment, Chartis testing, or valve deployment. The third was immediately after completion of valve deployment, including any repositioning or valve exchange, under GA. The fourth was after emergence and extubation, once awake, spontaneously breathing, and hemodynamically re‐stabilized.

### 2.7 Data Handling and Analysis

Given the physiological case‐series design and small sample size, analyzes were descriptive with patient‐level reporting. No inferential testing was performed. The primary within‐patient comparison was the paired change temporally associated with valve deployment under GA, comparing the pre‐deployment acquisition with the immediate post‐deployment acquisition. A secondary paired comparison evaluated the change between the awake pre‐induction acquisition and the post‐extubation acquisition once awake and stable. The change from pre‐induction to the pre‐deployment general anesthesia acquisition was used to contextualize the effects of induction and controlled ventilation.

Cardiac output (CO) was estimated using the indirect Fick principle with assumed oxygen consumption derived from the Sykes formula; arterial and mixed venous blood were used to derive oxygen content using standard equations. Prespecified derived variables were then calculated, including transpulmonary gradient (TPG, mean PA pressure [mPAP] − PCWP), pulmonary vascular resistance (PVR, TPG/CO; Wood units), stroke volume (SV, CO/heart rate), and pulmonary arterial compliance (PAC, SV/PA pulse pressure). Given the use of assumed oxygen consumption, CO and CO‐derived indices were treated as estimates, and interpretation focused on within‐patient paired changes.

## 3 Results

### 3.1 Participant Characteristics

Four consecutive patients underwent EBV implantation with peri‐procedural invasive hemodynamic assessment. All had severe emphysema on CT imaging, severe airflow obstruction (FEV_1_ 30%–40% predicted), marked hyperinflation (residual volume 175%–285% predicted), and reduced gas transfer (TLCO 34%–44% predicted) (Table 1). All were classified as American Society of Anaesthesiologists (ASA) physical status IV (American Society of Anaesthesiologists classification), indicating a very high‐risk procedural cohort. Symptom burden and functional limitation were substantial (modified Medical Research Council [mMRC] ≥ 3; performance status 2–3), and echocardiography suggested at least intermediate probability of pulmonary hypertension in three patients (Table 1).

**Table 1:** Baseline clinical and physiological characteristics of patients undergoing endobronchial valve therapy.

Metric	Subject 1	Subject 2	Subject 3	Subject 4
Age	70 s	50 s	70 s	70 s
Sex	Male	Male	Female	Male
CT emphysema severity	Severe	Severe	Severe	Severe
Airflow obstruction (severity, FEV_1_% predicted)	Severe (30)	Severe (37)	Severe (35)	Severe (40)
Hyperinflation (severity, RV % predicted)	Marked (203)	Marked (285)	Marked (175)	Marked (229)
TLCO (% predicted)	Severely reduced (34)	Moderately reduced (44)	Severely reduced (36)	Moderately reduced (42)
6MWD (severity, m)	Marked limitation (192)	Preserved (370)	Marked limitation (190)	Mild‐moderate limitation (340)
CAT score	High impact (26)	High impact (27)	High impact (28)	High impact (22)
mMRC dyspnea grade	4	4	3	3
Performance status	3	3	2	2
ASA grade	IV	IV	IV	IV
PH category on echocardiography	High	Intermediate	Intermediate	Mild

*Note:* Values are shown as a severity category with the measured value in parentheses where applicable. 6MWD categories were defined using BODE distance strata and CAT impact bands using standard thresholds; dyspnea was graded using the mMRC scale and functional limitation by performance status. TLCO is reported as % predicted. BODE distance strata: preserved ≥ 350 m; mild–moderate limitation 250–349 m; marked limitation 150–249 m; very severe limitation < 150 m. CAT bands: low 0–9; medium 10–20; high 21–30; very high 31–40.

Abbreviations: ASA = American Society of Anaesthesiologists (physical status classification), CAT = COPD Assessment Test, mMRC = modified Medical Research Council, RV = residual volume, TLCO = transfer factor for carbon monoxide, 6MWD = 6‐min walk distance.

### 3.2 EBV Intervention

One patient underwent left lower lobe treatment (five valves), one underwent left upper lobe treatment, including the lingula (five valves), and two underwent combined right upper and right middle lobe treatment (five and seven valves, respectively). One procedure required an intra‐procedural valve exchange to achieve an adequate seal. Post‐implantation chest radiography demonstrated complete target‐lobe atelectasis in all patients. No pneumothorax occurred.

In one patient, continuous PA pressure monitoring during valve deployment demonstrated an initial fall in mPAP, followed by re‐elevation, prompting valve exchange. After replacement, the reduction in mPAP was sustained.

### 3.3 Invasive Hemodynamic Assessment

At the pre‐induction baseline, all four patients met hemodynamic criteria for PH (mPAP > 20 mmHg) with a pre‐capillary profile, defined by PCWP ≤ 15 mmHg and estimated pulmonary vascular resistance (PVR) > 2 Wood units in all cases (Table 2, Figure 1). Arterial oxygen was preserved and comparable during paired intra‐anesthetic acquisitions (Supporting Information S1: Table 3). At the final post‐extubation timepoint, once awake and hemodynamically stable, three patients no longer met hemodynamic criteria for PH (mPAP < 20 mmHg) (Table 3). In the remaining patient, PH persisted (mPAP 24 mmHg) with elevated PCWP (19 mmHg) and low estimated PVR (0.3 Wood units), consistent with an isolated post‐capillary profile.

**Table 2:** Baseline invasive pulmonary hemodynamics and hemodynamic classification prior to general anesthesia.

	Subject 1	Subject 2	Subject 3	Subject 4
mPAP (mmHg)	38	21	33	33
PCWP (mmHg)	13	7	9	15
TPG (mmHg)	25	14	24	18
PVR (Wood units)	6.9	3.4	5.3	4.8
Classification of pulmonary hypertension	Pre‐capillary	Pre‐capillary	Pre‐capillary	Pre‐capillary

*Note:* Values represent pre‐induction awake baseline measurements. TPG was calculated as mPAP minus PCWP. Hemodynamic classification was assigned using contemporary criteria. Pulmonary hypertension was defined as mPAP > 20 mmHg. A pre‐capillary profile required PCWP ≤ 15 mmHg and PVR > 2 WU.

Abbreviations: GA = general anesthesia, mPAP = mean pulmonary artery pressure, PCWP = pulmonary capillary wedge pressure, PVR = pulmonary vascular resistance, TPG = transpulmonary gradient.

**Figure 1:**
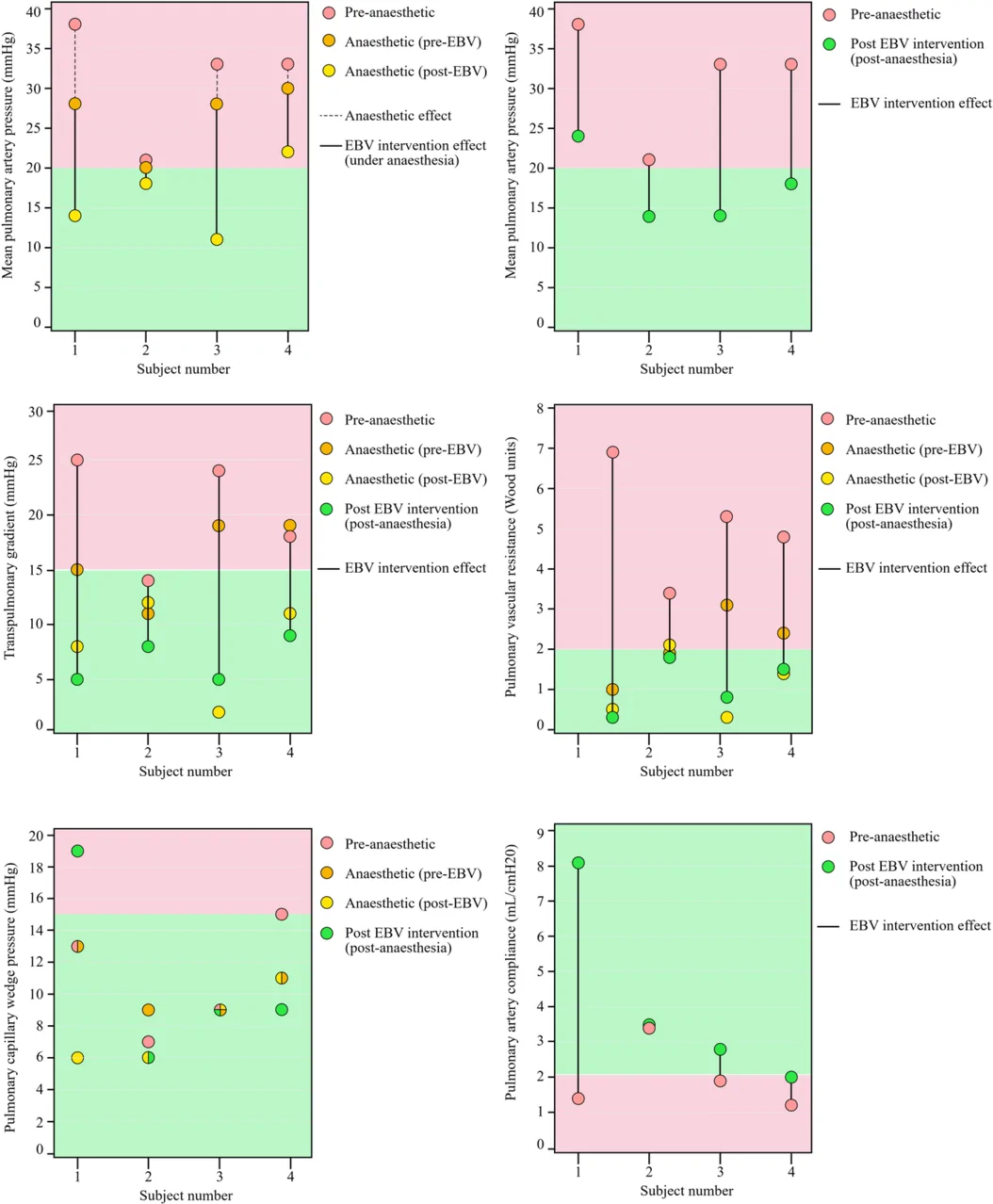
Acute pulmonary hemodynamic changes during endobronchial valve implantation in advanced emphysema with pre‐capillary pulmonary hypertension. EBV = endobronchial valve, mPAP = mean pulmonary artery pressure, PCWP = pulmonary capillary wedge pressure, PVR = pulmonary vascular resistance, TPG = transpulmonary gradient. Caption. Individual paired RHC measurements in four patients undergoing EBV implantation. Pre‐anesthetic, intra‐anesthetic pre‐EBV, intra‐anesthetic post‐EBV, and post‐anesthetic (awake) values are shown where available; connectors indicate within‐patient change (dashed, anesthetic effect; solid, EBV effect). Background shading indicates prespecified cut‐offs: mPAP 20 mmHg, TPG 15 mmHg, PVR 2 Wood units, PCWP 15 mmHg, and PAC 2 mL/mmHg.

**Table 3:** Post‐extubation invasive pulmonary hemodynamics and hemodynamic classification once awake and hemodynamically stable.

	Subject 1	Subject 2	Subject 3	Subject 4
mPAP (mmHg)	24	14	14	18
PCWP (mmHg)	19	6	9	9
TPG (mmHg)	5	8	5	9
PVR (Wood units)	0.3	1.8	0.8	1.5
Classification of pulmonary hypertension	Isolated post‐capillary	No PH	No PH	No PH

*Note:* Values represent the final post‐extubation measurements obtained once awake, spontaneously breathing, and hemodynamically stable. Hemodynamic interpretation uses the same criteria as Table 2. ‘Isolated post‐capillary PH’ indicates an elevated PCWP with low PVR, while ‘No PH’ indicates mPAP not meeting hemodynamic criteria for PH.

Abbreviations: mPAP = mean pulmonary artery pressure, PCWP = pulmonary capillary wedge pressure, PVR = pulmonary vascular resistance, TPG = transpulmonary gradient.

In the prespecified GA comparison (pre‐valve to post‐valve), mPAP decreased in all four patients, with within‐patient reductions ranging from 10% to 61%. In the awake comparison (pre‐induction to post‐extubation once awake and stable), mPAP also fell in all four patients (33%–58%), with post‐extubation mPAP below 20 mmHg in three patients (14, 14, and 18 mmHg).

Estimated PVR showed substantial reductions (Figure 1). From GA pre‐valve to post‐valve, PVR decreased in three patients (42%–90%) and increased modestly in one (11%). From pre‐induction to post‐extubation, PVR decreased in all four patients (47%–96%), yielding post‐extubation values of 0.3–1.8 Wood units.

Across the GA pre‐valve to post‐valve interval, TPG decreased from 11 to 19 mmHg to 2–12 mmHg. Three patients demonstrated reductions of 42%–89%, while one showed a small increase of 9%. In the awake comparison, TPG decreased in all four patients, falling from 14 to 25 mmHg pre‐induction to 5–9 mmHg post‐extubation, corresponding to within‐patient reductions of 43%–80%.

PCWP was comparatively stable during GA, remaining unchanged in two patients and decreasing by 33%–54% in two (Figure 1). After extubation, PCWP varied more widely, ranging from a 40% decrease to a 46% increase across patients. PAC increased in all four patients, rising from 1.2 to 3.4 mL/mmHg at baseline to 2.0–8.1 mL/mmHg post‐extubation, with absolute increases of 0.1–6.7 mL/mmHg.

## 4 Discussion

### 4.1 Key Findings

In this four‐patient prospective physiological case series, real‐time invasive hemodynamic assessment during EBV implantation identified acute within‐patient reductions in pulmonary vascular load in selected patients with advanced emphysema who met baseline hemodynamic criteria for pre‐capillary PH. The paired pre‐valve to post‐valve comparison under GA showed a fall in mPAP in all four patients and a reduction in TPG in most, a pattern compatible with an acute reduction in RV afterload temporally aligned with valve deployment. After extubation, once awake and hemodynamically stable, three patients no longer met hemodynamic criteria for PH, and PAC increased in all four. These findings are descriptive and hypothesis‐generating; they indicate that an immediate hemodynamic response may occur in selected patients, but they do not establish durability, prognostic significance, or a change in clinical eligibility thresholds.

### 4.2 Context and Rationale

EBV therapy is established as a bronchoscopic lung volume reduction strategy in carefully selected patients with severe emphysema, marked hyperinflation and absent collateral ventilation, supported by multicentre randomized trials demonstrating clinically meaningful improvements in lung function, exercise capacity, and health status [5, 6, 8, 10]. The question addressed by the present series is therefore not whether EBV is effective in suitable emphysema phenotypes, but how pulmonary hemodynamics behave in patients in whom PH is suspected or present, a subgroup that is frequently approached with caution [15].

The question is clinically relevant because PH in COPD is common, prognostically adverse, and mechanistically heterogeneous [16]. Invasive hemodynamics remain central to classification, particularly to distinguish predominantly pulmonary vascular phenotypes, characterized by elevated pulmonary pressures with normal left‐sided filling pressures, from phenotypes driven primarily by raised left‐sided filling pressures [17, 18]. More severe pulmonary vascular phenotypes in COPD are less frequent but carry a higher risk and often influence eligibility and procedural risk discussions for lung volume reduction interventions [15, 19].

Patients with significant PH have also been under‐represented in the pivotal EBV evidence base. Major EBV trials and device eligibility criteria have commonly excluded significant PH, either through hemodynamic thresholds or by excluding patients with “known” or “severe” PH and cor pulmonale [9, 10, 20]. Contemporary BLVR consensus guidance reflects this uncertainty, recommending caution when PH is present and advising against proceeding when it is severe [15, 21]. The present series, therefore, provides preliminary physiological observations relevant to an evidential gap, rather than data that should alter practice.

### 4.3 Mechanistic Considerations

A plausible explanation for the immediate hemodynamic changes observed is that EBV therapy may alter pulmonary vascular load through rapid changes in lung mechanics and regional gas exchange, rather than by modifying established pulmonary vascular remodeling (Figure 2) [13]. In this series, the paired GA comparison demonstrated reductions in mPAP accompanied by falls in TPG in most patients, while wedge pressure was stable overall. This pattern is compatible with reduced pulmonary vascular load at the time of deployment, but the mechanistic interpretation remains speculative in a four‐patient uncontrolled series.

**Figure 2:**
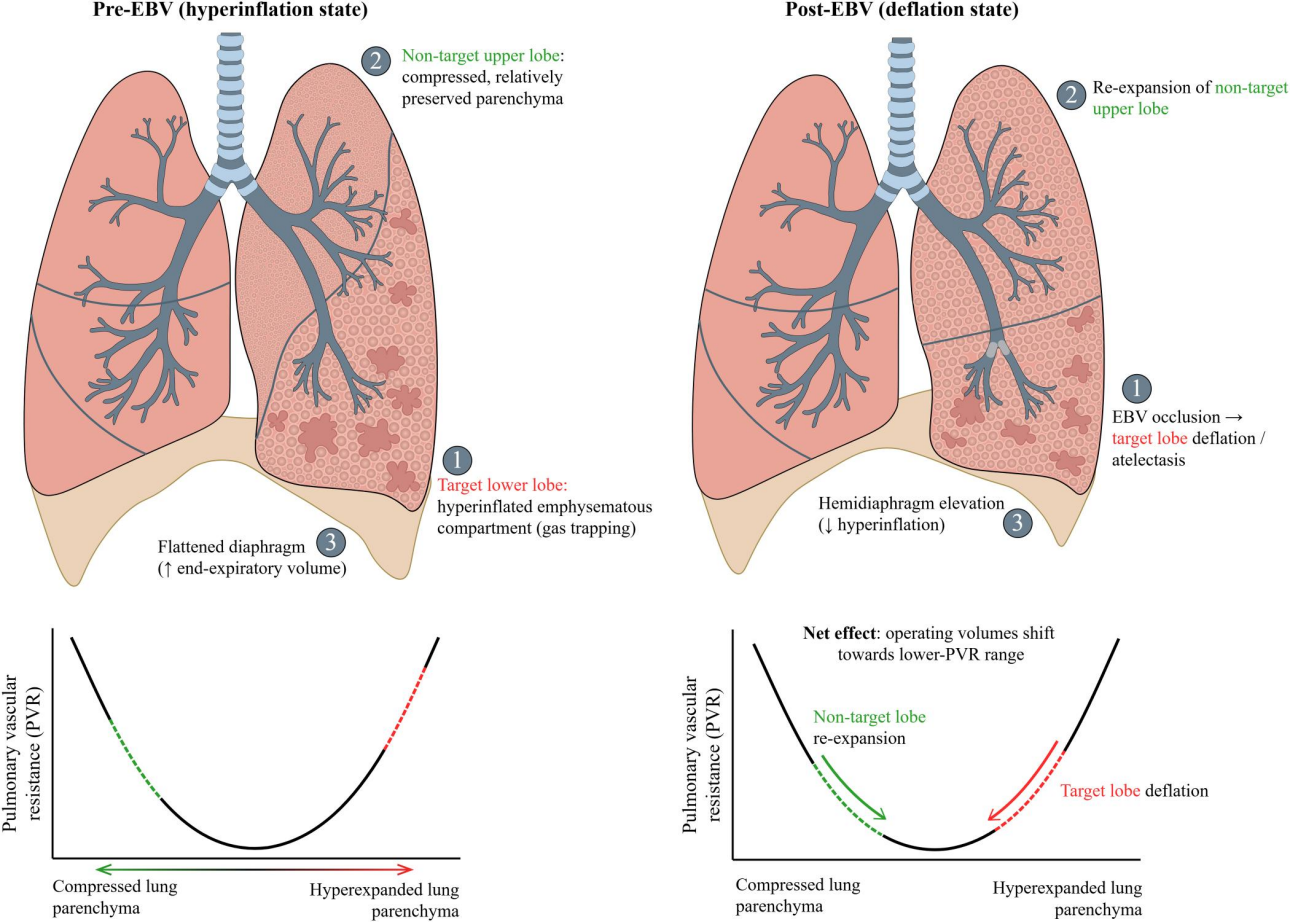
Mechanistic schematic of endobronchial valve‐induced lung deflation and its conceptual link to pulmonary vascular load. EBV = endobronchial valve, FRC = functional residual capacity, PVR = pulmonary vascular resistance. Caption. Upper panels: before EBV, a hyperinflated emphysematous lower lobe compresses the upper lobe and depresses the hemidiaphragm; after EBV, target‐lobe deflation permits upper‐lobe re‐expansion and diaphragm elevation. Lower panels: schematic PVR–lung volume relationship illustrating regional operating‐volume shifts away from high‐volume, high‐resistance conditions toward lower‐resistance volumes.

Mechanistically, deflation of hyperinflated lung regions could relieve microvascular compression and reduce the intrathoracic pressure burden that contributes to RV loading, while redistribution of ventilation away from severely diseased units may attenuate hypoxic pulmonary vasoconstrictor drive [22, 23]. However, systemic oxygenation was high and comparable during the paired intra‐anesthetic measurements, making progressive correction of global hypoxemia an unlikely explanation for the immediate change. The increase in PAC at the final awake timepoint is directionally concordant with reduced pulsatile afterload, while recognizing that compliance is a derived measure dependent on cardiac output estimation.

The heterogeneity in wedge pressure observed after extubation, including one patient with an isolated post‐capillary profile, is also physiologically plausible in severe emphysema. Transitions between ventilatory states, changes in intrathoracic pressure, and peri‐procedural volume sensitivity can substantially influence left‐sided filling pressures in susceptible patients. In this case, co‐existing left‐heart substrate on echocardiography (moderate mitral regurgitation and regional LV dysfunction) provides a plausible explanation for the post‐intubation rise in PCWP. This variability warrants caution when interpreting post‐extubation hemodynamic classification in a high‐risk emphysema cohort [24].

### 4.4 Novelty and Relationship to Prior Work

Prior literature has reported cardiopulmonary changes after EBV therapy, but hemodynamic evidence has generally been indirect or derived from delayed assessments [25, 26]. Echocardiographic studies have described changes in RV function following endoscopic lung volume reduction, and prospective work has shown that reducing hyperinflation with EBV can improve cardiac preload, contractility, and output; however, these approaches cannot directly resolve immediate pulmonary pressures, gradients, or resistance at implantation [25].

A small invasive pilot series in patients with established PH and other observational work reported hemodynamic changes months after EBV implantation or in association with target‐lobe volume reduction [27, 28, 29]. The present series adds intra‐procedural temporal resolution and invasive phenotyping, with paired measurements obtained before and after valve deployment within the same anesthetic state. The pre‐valve acquisition was also performed before bronchoscopic assessment, including Chartis testing, reducing the likelihood that the observed paired GA change reflected transient effects of lobar occlusion testing rather than implantation itself.

### 4.5 Anesthesia and State Effects

The hemodynamic shift observed with induction and controlled ventilation in this cohort is physiologically credible and reinforces the importance of procedural state when interpreting pulmonary pressures in severe emphysema. Spontaneous breathing can generate large intrathoracic pressure swings and dynamic hyperinflation, whereas GA and controlled positive‐pressure ventilation can alter lung volume, respiratory mechanics, oxygenation, acid–base status, venous return, and pulmonary vascular tone [30]. Accordingly, comparisons across awake and anaesthetized states should be interpreted as contextual rather than causal. By incorporating a pre‐valve acquisition under GA, the paired GA comparison provides the most direct estimate of the immediate valve‐associated change in this design, but residual confounding by anesthetic and ventilatory conditions cannot be excluded.

### 4.6 Clinical Implications

Clinically, these observations should be interpreted conservatively. They do not support expanding EBV indications to patients with PH, lowering current safety thresholds, or proceeding in severe or fixed pulmonary vascular disease outside established expert guidance. At most, the data suggest that pulmonary vascular load in some hyperinflated emphysema patients may be labile around the time of intervention and that invasive phenotyping may help distinguish pre‐capillary from post‐capillary physiology when management decisions are affected. Intra‐procedural RHC also provided immediate physiological feedback in one patient with suspected inadequate valve sealing, but this observation is anecdotal and should be viewed as proof‐of‐concept for future study rather than a recommendation for routine monitoring.

## 5 Limitations

This study has important limitations. The sample comprises only four patients from a single center, with no control group; all results are descriptive and hypothesis‐generating. Pressures under GA were sampled at end‐expiration without a protocolised end‐expiratory pause, introducing potential respiratory variability. Central venous pressure was not recorded at every hemodynamic acquisition; therefore, progressive preload change cannot be fully excluded as a contributor. CO was estimated using the indirect Fick method with assumed oxygen consumption and without thermodilution, limiting confidence in absolute CO and derived indices such as PVR and compliance, particularly across physiological states. For this reason, interpretation should prioritize directly measured pressures and TPG and focus on within‐patient paired changes. The post‐extubation timepoint reflects a re‐stabilized awake state rather than a fixed recovery interval, and peri‐procedural factors such as fluid balance, analgesia and sympathetic tone may influence wedge pressure and systemic hemodynamics.

## 6 Future Directions

Future studies should evaluate the durability of these findings by repeating invasive hemodynamic assessments several months after EBV implantation to determine whether acute changes persist and whether early hemodynamic responses predict long‐term clinical and imaging outcomes. Larger cohorts with standardized ventilatory conditions, direct CO assessment, and linkage of acute hemodynamic changes to target‐lobe volume reduction, RV function, gas exchange, and patient‐centered outcomes will be required before any change in patient‐selection thresholds can be considered.

## 7 Conclusion

In this four‐patient prospective case series, invasive hemodynamic assessment performed in real time during EBV implantation was feasible and identified rapid within‐patient reductions in pulmonary artery pressure and pulmonary vascular load in selected patients with advanced emphysema and baseline pre‐capillary pulmonary hypertension. These preliminary data raise the hypothesis that part of the pulmonary vascular load in some hyperinflated emphysema patients may be acutely modifiable during lung deflation. They should not be interpreted as evidence to expand EBV indications to patients with PH. Larger, standardized studies are required to determine the durability, safety, and prognostic significance of acute hemodynamic responses.

## Author Contributions

Conceptualisation: Vasileios Kouritas and Henry O'Connor. Methodology: Vasileios Kouritas, Henry O'Connor, Ciaran Grafton‐Clarke, and Pankaj Garg. Investigation: Vasileios Kouritas, James Harrison, Henry O'Connor, and Ciaran Grafton‐Clarke. Data curation: Ciaran Grafton‐Clarke and Henry O'Connor. Formal analysis: Ciaran Grafton‐Clarke and Henry O'Connor. Writing – original draft: Ciaran Grafton‐Clarke. Writing – review and editing: Ciaran Grafton‐Clarke, James Harrison, Della Mantle, Gareth Matthews, Pankaj Garg, Vasileios Kouritas and Henry O'Connor. Supervision: Pankaj Garg, Vasileios Kouritas, and Henry O'Connor. Project administration: Ciaran Grafton‐Clarke.

## Ethics Statement

This work was conducted as a prospective physiological case series embedded within routine clinical care. All procedures, including endobronchial valve implantation and invasive hemodynamic assessment, were performed for clinical indications. No research‐specific interventions were undertaken. Written informed consent was obtained from all participants for the procedure and for the use of anonymized clinical and hemodynamic data for analysis and publication.

## Conflicts of Interest

The authors declare no conflicts of interest.

## Guarantor

The corresponding author acts as guarantor.

## Supporting information


Supporting File
