# Supplementary material for: Real‐Time Invasive Hemodynamic Changes During Endobronchial Valve Therapy in Advanced Emphysema: A Prospective Case Series
Source: Pulm Circ. 2026 Nov 5;16(4):e70451. doi: 10.1002/pul2.70451 (PMC13737470; doi:10.1002/pul2.70451)
Supplement: Supplementary file 1 [file PUL2-16-e70451-s001.docx]

**Supplementary Table 1.** Induction and reversal of general anaesthesia.

| **Parameter** | **Comment** |
| --- | --- |
| Induction | Propofol and alfentanil |
| Paralytic agent | Atracurium |
| Reversal agent | Neostigmine/Glycopyrronium |
| Analgesia | Intravenous paracetamol |
| Antibiotics | Intravenous co-amoxiclav (subject 2-4) and intravenous teicoplanin (subject 1) |

**Supplementary Table 2.** Mechanical ventilation settings.

| **Ventilator setting** | **Comment** |
| --- | --- |
| Respiratory rate (breaths per minute) | 12 |
| Positive-end-expiratory pressure (cmH_2_O) | 5 |
| Peak airway pressures (cmH_2_O) | 18-22 |
| Fraction of inspired oxygen (FiO_2_) | 0.5 – 0.7 |

**Supplementary Table 3.** Oxygenation at each haemodynamic acquisition

| **Measurement** | **Subject 1** | **Subject 2** | **Subject 3** | **Subject 4** |
| --- | --- | --- | --- | --- |
| Pre-GA SaO_2_ | 98 | 98 | 95 | 95 |
| Pre-GA SvO_2_ | 69 | 70 | 71 | 59 |
| GA pre-valve SaO2 (%) | 100 | 100 | 100 | 100 |
| GA pre-valve SvO2 (%) | 93 | 80 | 82 | 83 |
| GA post-valve SaO_2_ (%) | 100 | 98 | 100 | 100 |
| GA post-valve SvO_2_ (%) | 93 | 70 | 82 | 83 |
| Post-extubation SaO_2_ (%) | 100 | 92 | 86 | 98 |
| Post-extubation SvO_2_ (%) | 94 | 66 | 69 | 76 |

**Abbreviations.** GA, general anaesthesia; SaO₂, arterial oxygen saturation; SvO₂, mixed venous oxygen saturation.

**Caption**. Oxygen saturation values recorded at each prespecified haemodynamic acquisition. Intra-anaesthetic measurements were performed under controlled ventilation with FiO₂ maintained at 0.5–0.7 (Supplementary Table 2), and FiO₂ was kept constant within each patient between the paired intra-anaesthetic pre- and post-valve acquisitions. Post-extubation values were recorded after emergence once patients were awake and spontaneously breathing and should not be interpreted as being obtained under the same FiO_2_ as the paired intra-anaesthetic measurements.
